# Replication and Meta-Analysis of Common Gene Mutations in *TTF1* and *TTF2* with Papillary Thyroid Cancer

**DOI:** 10.1097/MD.0000000000001246

**Published:** 2015-09-11

**Authors:** Yan Gao, Fei Chen, Shuli Niu, Shiyu Lin, Suping Li

**Affiliations:** From the Department of Nuclear Medicine of Affiliated Hospital of North Sichuan Medical College, Nanchong 637000, Sichuan Province, China; and Sichuan Key Laboratory Medical Imaging, Nanchong 637000, Sichuan Province, China.

## Abstract

Papillary thyroid cancer (PTC), one of the most common malignant thyroid tumors, exits widely in the thyroid of adolescents. Thyroid transcription factor 1 (*TTF1*) and 2 (*TTF2*) were thyroid-specific transcription factors, and regulated expression of the thyroid-specific genes. Hence, the aim of the present study was to evaluate the correlation between gene variants of *TTF1* and *TTF2* and the risk of PTC in Chinese population.

Two tagging single-nucleotide polymorphisms (tSNPs) on *TTF1* and *TTF2* were selected and genotyped by matrix-assisted laser desorption/ionization time-of-flight (MALDITOF) mass spectrometry in a hospital-based case-control study of 297 PTC patients and 594 healthy controls. Furthermore, a meta-analysis of the association between *TTF1* and *TTF2* and PTC risk was also performed.

We found that the rs944289 on the *TTF1* was significantly associated with increased PTC risk (TT vs CC, OR = 1.53, 95% CI = 1.05–2.24; CT + TT vs TT, OR = 1.34, 95% CI = 1.00–1.79; T vs C, OR = 1.27, 95% CI = 1.04–1.55). Similarly, the rs965513 on the *TTF2* can also elevate the risk of PTC significantly (GA vs GG, OR = 1.67, 95% CI = 1.07–2.59; AA+GA vs AA, OR = 1.37, 95% CI = 1.09–1.82; A vs G, OR = 1.29, 95% CI = 1.05–1.59). Furthermore, results of stratified analysis revealed that the risk effects of rs944289 and rs965513 were more overpowering in the subgroups of patients with MNG, as well as subjects without metastasis. Results of meta-analysis from the previous study and our new data indicated that variants of rs944289 and rs965513 might be the genetic susceptible factors both in Asians and Caucasians.

We get the conclusion that mutations of *TTF1* and *TTF2* are significantly associated with an increasing risk of PTC in Chinese. However, more detailed investigations and further large-scale studies on genetic functions to provide more conclusive and accurate evidence are required in the future.

## INTRODUCTION

Thyroid cancer (TC) is one of the most familiar endocrine malignancies, which accounts for 1% of all tumors and 0.2% of cancer deaths, and its incidence grows rapidly in recent years.^1^ At present, the incidence ratio of TC in all races is 18.2 and 6.2 per 100,000 females and males respectively according to the latest global cancer statistics during 2006–2010.^2^ TC has 4 main subtypes: papillary (PTC), medullary (MTC), follicular (FTC), and anaplastic thyroid cancer (ATC). Papillary thyroid carcinoma (PTC), which is the main subtype of thyroid tumor and occurred usually in the women in the age group from 20 to 50 years, accounts for 75 to 85% among all TC patients.^3^ PTC is a relatively indolent tumor and 25-years survival rate is up to 95% when the patients accept overall and appropriate treatments.^4^ However, it is reported that the occurrence of PTC in the last decades increased sequentially and intensely in the world.^5^ It aroused people's attention widely because of its increasing incidence. Generally, the pathological mechanism of PTC is considered as a complex process, which is caused mainly by the combined action of environmental and genetic factors.^6^ Ionizing radiation, smoking, drinking, and working environment can induce PTC.^7^ However, owing to its high heritability of PTC in recent years, genetic susceptibility and several relativity genes aroused people's attention widely.^8,9^

Thyroid transcription factor 1 (*TTF1*) and 2 (*TTF2*) are considered as the thyroid transcription factors (TTFs) collectively, which can encode the homeobox protein of transcription factors.^10^ They are expressed in several different tissues. Dysregulation and variants of *TTF1* and *TTF2* can cause many diseases, including cancers. The human *TTF1* protein, which contains 17 amino acids, was first found in the thyroid follicular cells, and then in the certain areas of brain and the lung subsequently.^11^ It is reported that *TTF1* could regulate a sequence of peripheral lung cells, which is called the terminal respiratory unit (TRU).^12^ Different intensities of *TTF1* were expressed in various stages of lung cancer.^13^ Approximately 70% adenocarcinomas also express *TTF1* differently in every stage and have the features of TRU to a certain degree.^13^ Meanwhile, Nettore et al found that a frameshift variant (c.493delC) in *TTF1* could advance risk of brain–thyroid–lung syndrome (BTLS).^14^ The study by Salvado and his colleagues discovered that variant (c.825delC) in *TTF1* had the significant association with Chorea.^15^ Similarly, *TTF2*, a gene of forkhead gene family, includes only one exon, which can encode 367-amino-acids with a highly conserved DNA-binding region called the forkhead domain.^16^ In humans, *TTF2* is expressed in many tissues, such as hair follicles, testis, and skin epidermis.^17^ Interestingly, *TTF1* and *TTF2* are only expressed together in the follicular epithelial cells of thyroid.^18^ These epithelial cells play an important role in the structure of follicular, which is the main cell type in thyroid tissue and has closest relationship with synthesis of thyroid hormones.^19^

Expression changes and/or gene variants of *TTF1* and *TTF2* have the relationship with PTC and non-PTC, although the decisive pathogenesis is still unclear.^20^ In the last decades, several studies found that *TTF1* and *TTF2* genetic polymorphisms might be positively related with the susceptibility of PTC.^21–27^ Meanwhile, a genome-wide association study (GWAS) in European is considered as a golden standard for identifying the association between genotype and phenotype of PTC. However, there are still some considerable argument about the association between gene polymorphisms of *TTF1* and *TTF2* and risk of PTC. As stated by McClellan and King, many if not most about gene mutation may be factually false results because the subjects of above studies mainly come from Europe in GWAS.^28^ Moreover, phenotypic heterogeneity, sample size, and ethnic diversity are all the restricted factors in the individual studies about the gene polymorphism and PTC risk. Further assessment is needed to validate the association between gene mutations of *TTF1* and *TTF2* and risk of PTC.

Considering the conflicting results on this study, a case-control study was performed to replicate previously studies on the association between *TTF1* and *TTF2* gene variations and PTC risk in a Chinese population first. Additionally, we conducted a meta-analysis of all available published studies and the present data to derive a relative precise evaluation about the relationship between common mutations of gene and susceptibility of PTC.

## METHODS

### Study Subjects

A case-control study was conducted to confirm the relationship between common mutations of *TTF1* and *TTF2* genes and susceptibility of PTC. Blood samples were derived from 297 newly diagnosed PTC patients and 594 healthy individuals between November 2010 and June 2014 from Affiliated Hospital of North Sichuan Medical College. All the recruited subjects were Han Chinese with no genetic relationship with each other, and all of them come from Eastern China, mainly containing Hangzhou, Jiangsu, Shanghai, and surrounding regions. PTC patients were confirmed by two thyroid pathologist independently through histopathological examination according to the World Health Organization standards. All patients and healthy individuals have no previous radiation exposure. None of the participants had history of other cancer and received chemotherapy or radiotherapy. The cases and controls were well matched for age and sex, with *P* value of 0.840, and 0.918, respectively. Clinicopathologic information were collected from electronic clinical and pathologic records, such as multiplenodular goiter (MNG), tumor size, extrathyroidal extension, multifocality, kinds of metastasis (node metastasis and distant metastasis), infiltration (intratumoral lymphocytic infiltration and peritumoral lymphocytic infiltration), and concomitant thyroid disease. Detailed clinical characteristics of the study subjects are summarized in Table 1. All participants included in the present study received the pre-tested questionnaire, which included the information and data about demographic data, smoking, drinking, family diseases history, and so on. The mission was done by experienced and trained interviewers. The study was approved by the ethical committee of Affiliated Hospital of North Sichuan Medical College. Written informed consents were acquired from all volunteers before the commencement of the research.

**TABLE 1:**
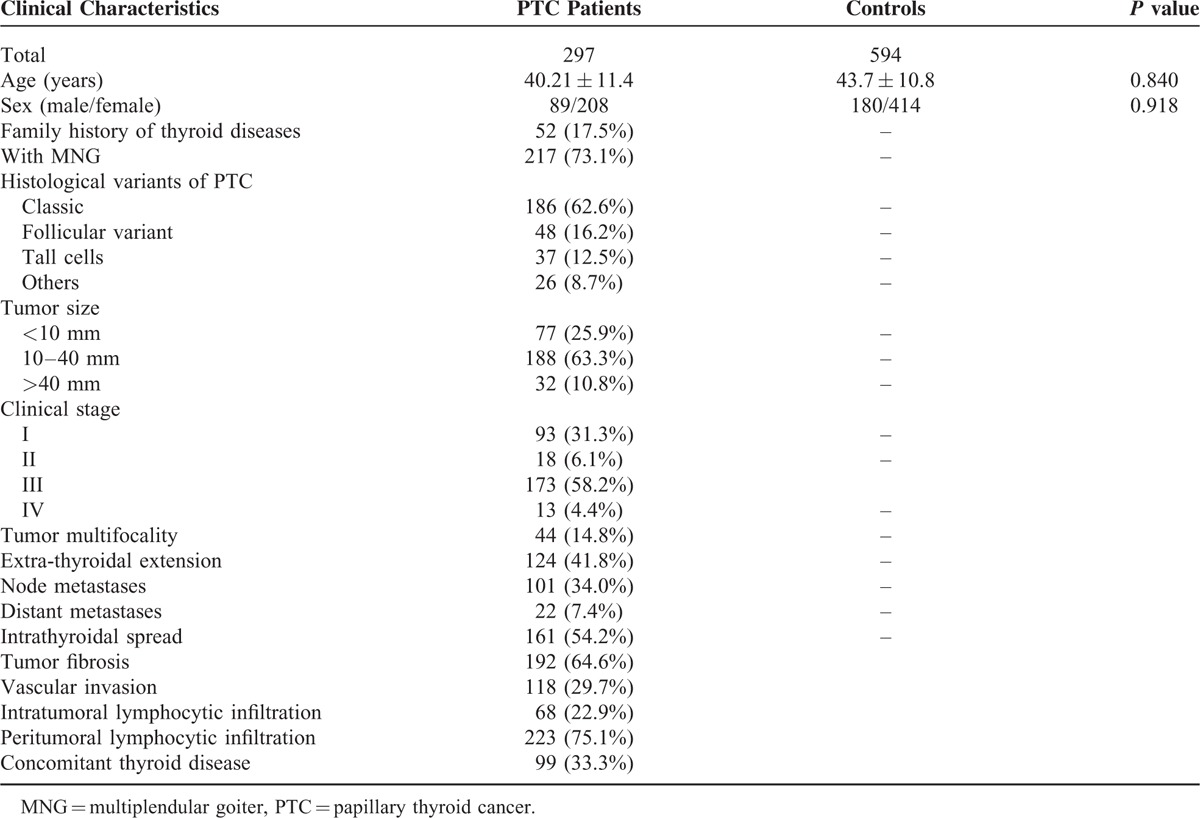
Comparison of PTC Patients and Controls by Selective Characteristics

### SNP Selection

Recently, GWAS was used in identifying the relationship between gene variants and risk of PTC in Europeans. The result revealed that two common variants (rs944289, located on 14q13.3; rs965513, situated on 9q22.33) could elevate the risk of PTC significantly.^29,30^ Meanwhile, many studies found variants of rs944289/rs965513 could positively elevate the risk of PTC significantly.^31–34^ Among the nearest genes existed in the block of linkage disequilibrium (LD) containing the two SNPs (rs944289/ rs965513) in GWAS are *TTF1*, and *TTF2*, respectively.^20^ Finally, two SNPs (rs944289/ rs965513) were selected.

### DNA Extraction and Genotyping

Genomic DNAs were extracted by Qiagen DNA blood kit (Qiagen, Hilden, Germany) from whole blood samples collected from all subjects according to the manufactures’ instruction. In summary, blood samples needed digestion with sodium dodecyl sulfate at 56°C for 12 h in the homothermic water bath. All studied genetic polymorphisms were analyzed using matrix-assisted laser desorption/ionization time-of-flight (MALDITOF) mass spectrometry (Sequenom, Inc.). Spectro DESIGNER software (Sequenom, Inc.) was used to design assays for the studied SNPs. The iPLEX assays were used to genotype all the SNPs. The amplification reaction was conducted in a bulk volume of 8 μl, including 800 mM of dNTP mix, 16 ng of genomic DNA, 2.6 mM MgCl_2_, 160 nM of amplification primer in each reaction, 0.8 units of HotStarTaq DNA Polymerase (Qiagen Inc.). All the primers for PCR amplification are showed as follows: rs944289: 5′-GGG GAA GCC AAG TGT AGG-3′ (forward); 5′-CTG TGC GGG GAC TGT TAA-3′ (reverse); rs965513: 5′- GAC AAA GGT AAT GAG TGG-3′ (forward); 5′-TTG TTA GCA TTG TGA GAA-3′ (reverse). The tubes were amplied according to the following PCR conditions: 15 min at 95°C for a single cycle of initial denaturation, 20 s for 45 cycles at 94°C of denaturation, 56°C annealing for 30 s, extension 60 s at 72°C, and 72°C for 3 min for the final extension. Primer extension reactions’ products were mounted on a pipetting system with 384-element chip nanoliter (Sequenom Inc.), and then analyzed on the microflex Series Benchtop MALDI-TOF Mass Spectrometer (Bruker Daltonik GmbH, Germany). Two skillful persons analyzed the data independently using the SpectroTyper software (Sequenom, Inc.).

### Statistical Analysis

For each SNP, Hardy–Weinberg equilibrium (HWE) was used to access in the control and case groups independently with chi-square test. In addition, the chi-square test was also used to analyze various types of genetic and allele frequencies of the SNPs in the control and the case groups. Meanwhile, we used the Student's *t*-test to differentiate demographic characteristics (such as sex, age, and family history of thyroid disease). Odds ratios (ORs) and 95% confidence intervals (CIs), which were calculated by unconditional logistic regression and multivariate logistic regression respectively, were used to assess the relationships between gene variations and risk of PTC. The significance levels for statistical tests were evaluated by two-tailed *P* value, and it is considered statistically significant if *P* value is <0.05. The selected SNPs come from highly credible genes reported previously as the pathogenic gene in PTC and were functional variants potentially. These provide a priori demonstration for their relationship, and amendments of multiple comparisons were not needed. GraphPad Prism 6.0 was used to generate the graphs.

In the meta-analysis, *I*^*2*^ and Cochran's Q-test were used to evaluate heterogeneity of Between-studies. When *I*^*2*^ < 50% and *P* ≥ 0.05 of the *Q*-test, we used the fixed effect model to pool the data; otherwise, the random effect model was adopted. The Z test was used to appraisal whether pooled OR had the statistical significance. Publication bias was estimated by funnel plots and Egger's linear regression test. STATA version 11.0 (Stata Corporation, College Station, TX) was used to perform all the statistical tests in the meta-analysis.

## RESULTS

### Sample Characteristics

In this study, we derived blood samples from 297 PTC patients (included 89 males and 208 females) and 594 healthy controls (consisted of 180 males and 414 females). As shown in Table 1, they appeared to be matched well on age and sex, with *P* = 0.840, and *P* = 0.918, respectively. The mean age of the subjects ranged from 27 to 59 years. Meanwhile, PTC patients were confirmed by two pathologists independently according to the World Health Organization standards. Microscopic features of PTC patients were evaluated as follows: tumor size (small: 25.9%, median: 63.3%, large 10.8%), clinical stage (37.4% at early stage and 62.6% at advanced stage), tumor multifocality (yes:14.8%; no:85.2%), kinds of extension (extrathyroidal: 41.8%, intrathyroidal: 58.2%), fibrosis (64.6%) and sclerosis (35.4%), vascular invasion (yes: 29.7%; no: 70.3%), and types of lymphocytic infiltration (intratumoral: 22.9%, peritumoral: 75.1%, and others: 2%). Histological variants of PTC (classic, follicular, tall cells, and others) were also scored and showed as percentage of each kind, with 62.6%, 16.2%, 12.5%, and 8.7% respectively. Additionally, MNG, which is one common concomitant thyroid diseases account for 73.1% of all PTC patients, was also recorded.

### Association Between Individual SNP and Susceptibility of PTC without Stratification

The risk of the two SNPs were examined from each specimen with OR (95% CI) and *P* value (the detailed relationship between gene mutation of rs944289/rs965513 and the risk of PTC are shown in Table 2). The result revealed that no significant difference in the heterozygous and recessive models of rs944289/rs965513 was observed between PTC patients and healthy individuals. For rs944289, compared with the homozygote CC genotype, a high PTC prevalence of the variant TT genotype was observed and found a significant increasing of PTC risk (OR = 1.53, 95% CI = 1.05–2.24). A marginally positive correction was also noticed in the dominant model (CT+TT vs TT, OR = 1.34, 95% CI = 1.00–1.79). Meanwhile, in allele frequency distribution, significant difference between the 2 groups was found as well (OR = 1.27, 95% CI = 1.04–1.55). For another SNP (rs965513), it can also elevate the risk of PTC significantly in the heterozygote model (GA vs GG, OR = 1.67, 95% CI = 1.07–2.59), dominant model (AA+GA vs AA, OR = 1.37, 95% CI = 1.09–1.82) and allele model (A vs G, OR = 1.29, 95% CI = 1.05–1.59).

**TABLE 2:**
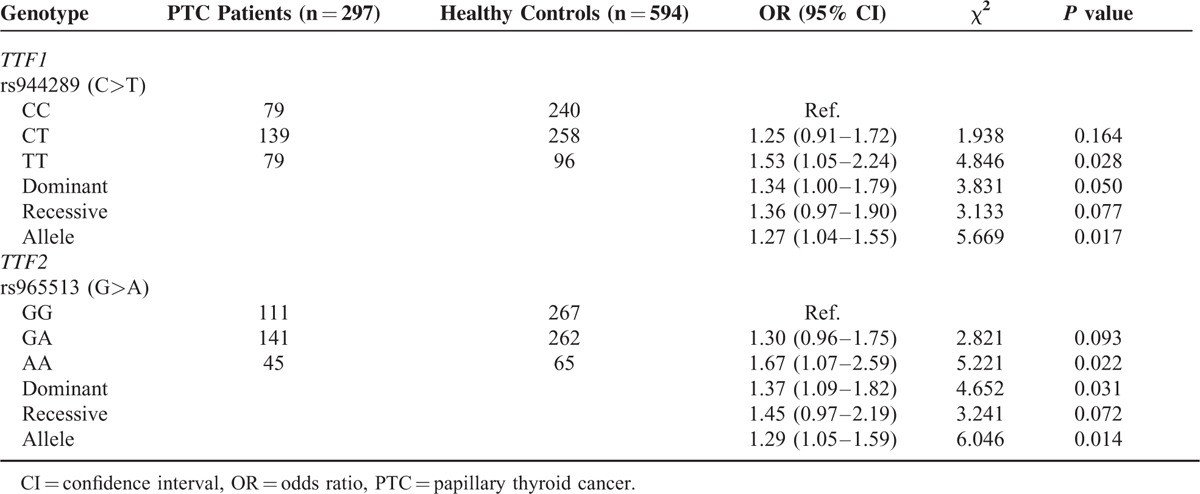
Associations Between 2 Polymorphisms of *TTF1* Gene and *TTF2* Gene and the Risk of Papillary Thyroid Cancer

### Association Between Individual SNP and Susceptibility of PTC with Stratification

In order to further evaluate the effect of mutation of rs944289/rs965513 on PTC occurrence, we did the stratification analysis by MNG and metastasis. As shown in Table 3, the association between mutation of rs944289/rs965513 and the risk of PTC appeared stronger in subgroups of patients with MNG or without metastasis. For rs944289, patients with MNG can elevate the risk of PTC significantly in the allele model (T vs C, OR = 1.72, 95% CI = 1.38–2.14) and in the dominant model (TC + TT vs CC, OR = 2.267, 95% CI = 1.58–3.23). However, a positive association was found between mutation of rs944289 and patients without MNG in the allele model, with OR of 1.45 (T vs C, 95% CI = 1.04–2.02), not in the dominant model. Meanwhile, polymorphism of rs944289 of patients without metastasis can increase the risk of PTC significantly, with OR of 2.07 (T vs C, 95% CI = 1.62–2.63), and OR of 2.35 (TC + TT vs CC, 95% CI = 1.58–3.47), respectively. Similarly, less robust relationships were found between rs965513 and patients with MNG under different models of inheritance (A vs G, OR = 1.32, 95% CI = 1.05–1.66; GA+AA vs GG, OR = 1.43, 95% CI = 1.04–1.97). Additionally, existence of minor A allele for rs965513 can increase the risk of metastasis significantly compared with wild-type allele G (OR = 1.42, 95% CI = 1.11–1.81).

**TABLE 3:**
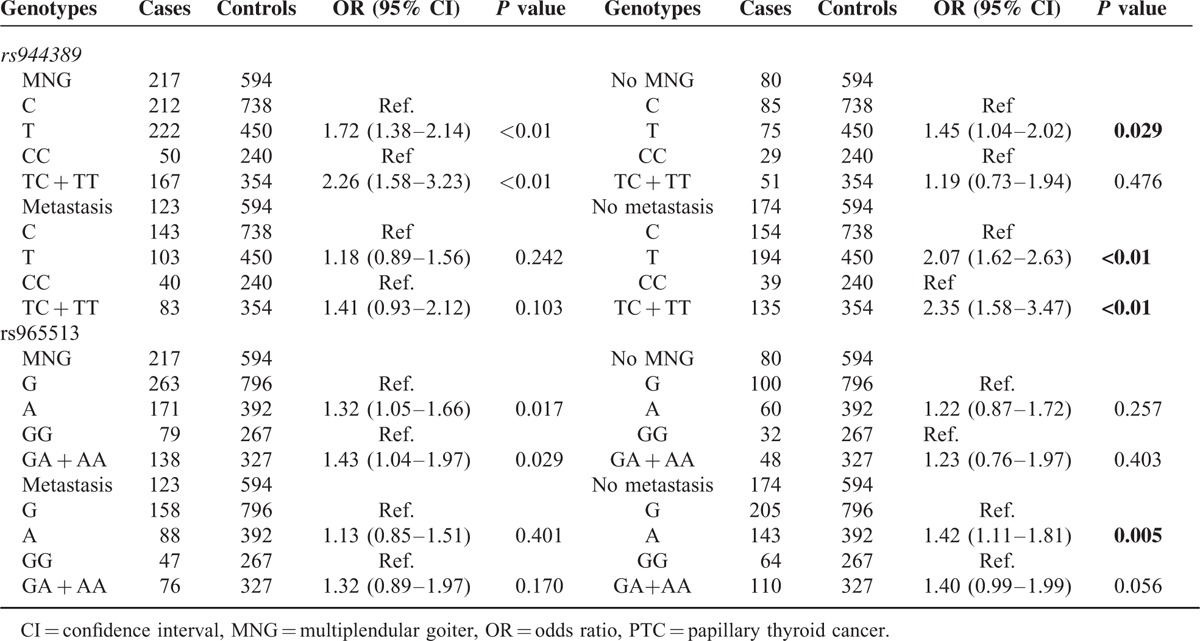
The Relationship Between Clinical Characteristics and the Genotypes of rs944389 and rs965513

### Meta-Analysis of the Association Between Individual SNP and PTC Risk

**FIGURE 1:**
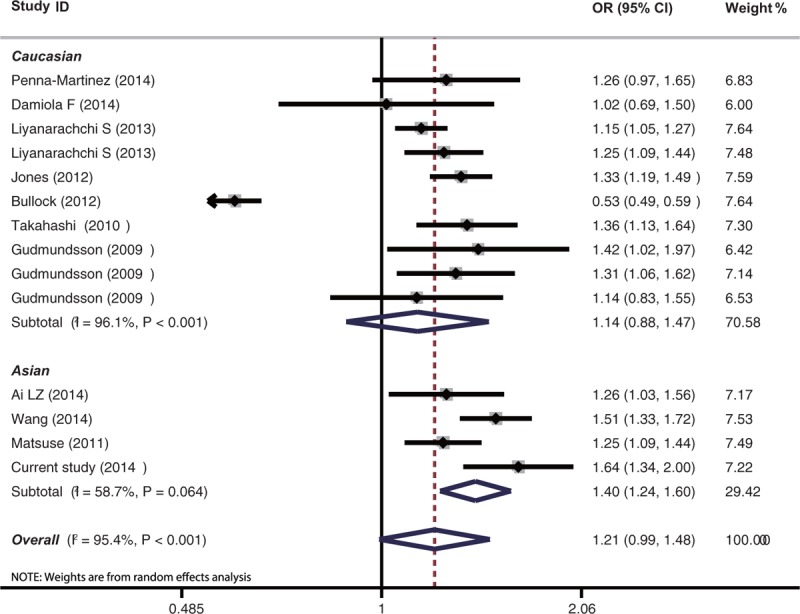
Forest plot for the meta-analysis of the association between rs944289 mutation and PTC.

**FIGURE 2:**
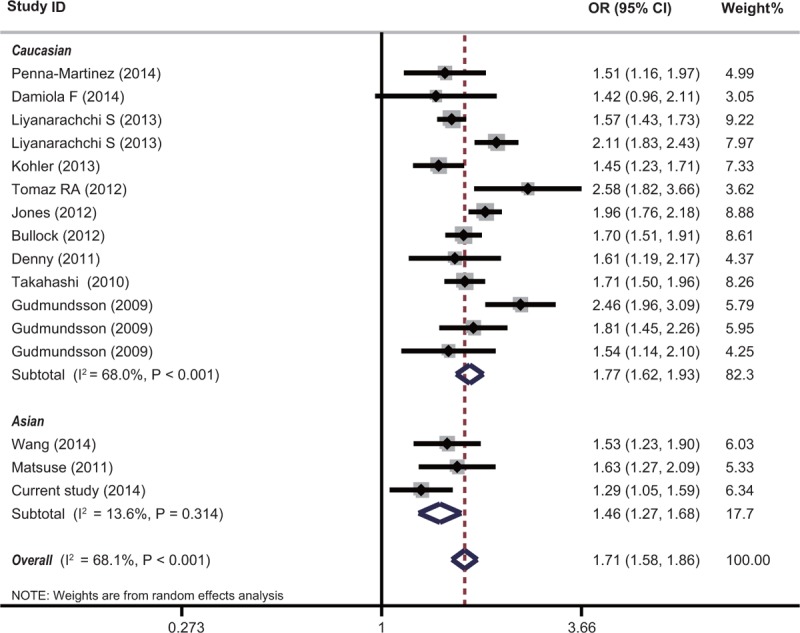
Forest plot for the meta-analysis of the association between rs965513 mutation and PTC.

We carried out meta-analysis through epitomizing the data of the present study and previous relevant studies, the detailed information of each study is tabulated in Table 4. The major results of meta-analysis of the relationship between rs944289/rs965513 and the risk of PTC are shown in Table 5. For rs944289 C/T polymorphism, it can elevate the risk of PTC significantly in the dominant model, homozygous model, and heterozygous model in the overall population. However, no association between rs944289 C/T variants and susceptibility of PTC in the allele model (as shown in Figure 1) and the recessive model were found. In the subgroup analysis by ethnicity, rs944289 mutation was significantly related with the risk of PTC in Asians in 5 models. Meanwhile, significant corrections were also found between rs944289 variant and PTC risk in Caucasian in the dominant model, homozygous model, and heterozygous model. As shown in Figure 2 and Table 5, 4 genotypes models and the allele model of rs965513 appeared to have significant associations in the overall population and subgroup analysis of ethnicity. Heterogeneity presented between studies and between subgroups with *P* < 0.001 for rs944289, but also for rs965513.

**TABLE 4:**
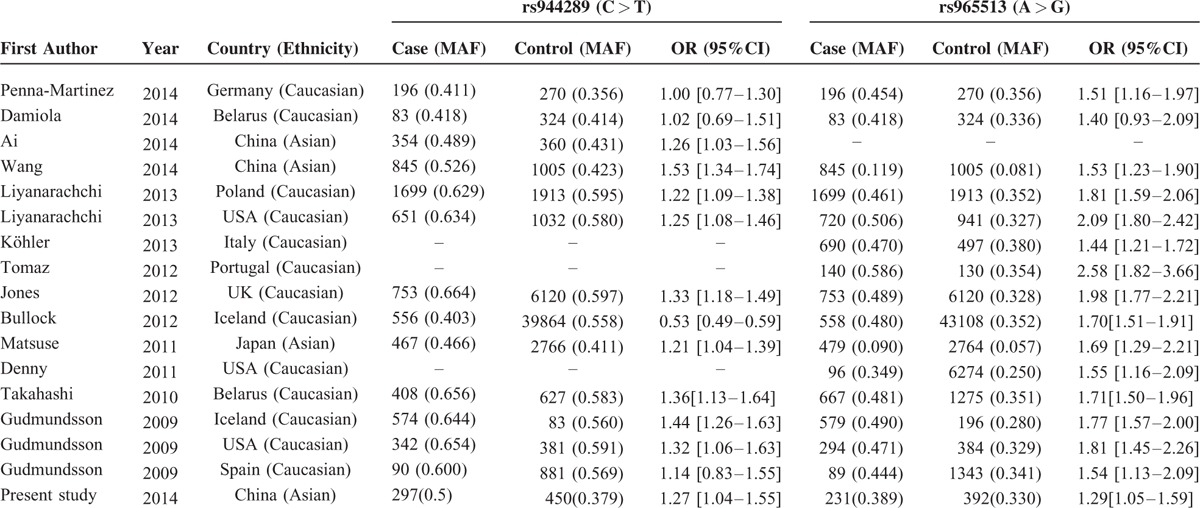
Main Results of the Studies Included in the Meta-Analysis

**TABLE 5:**
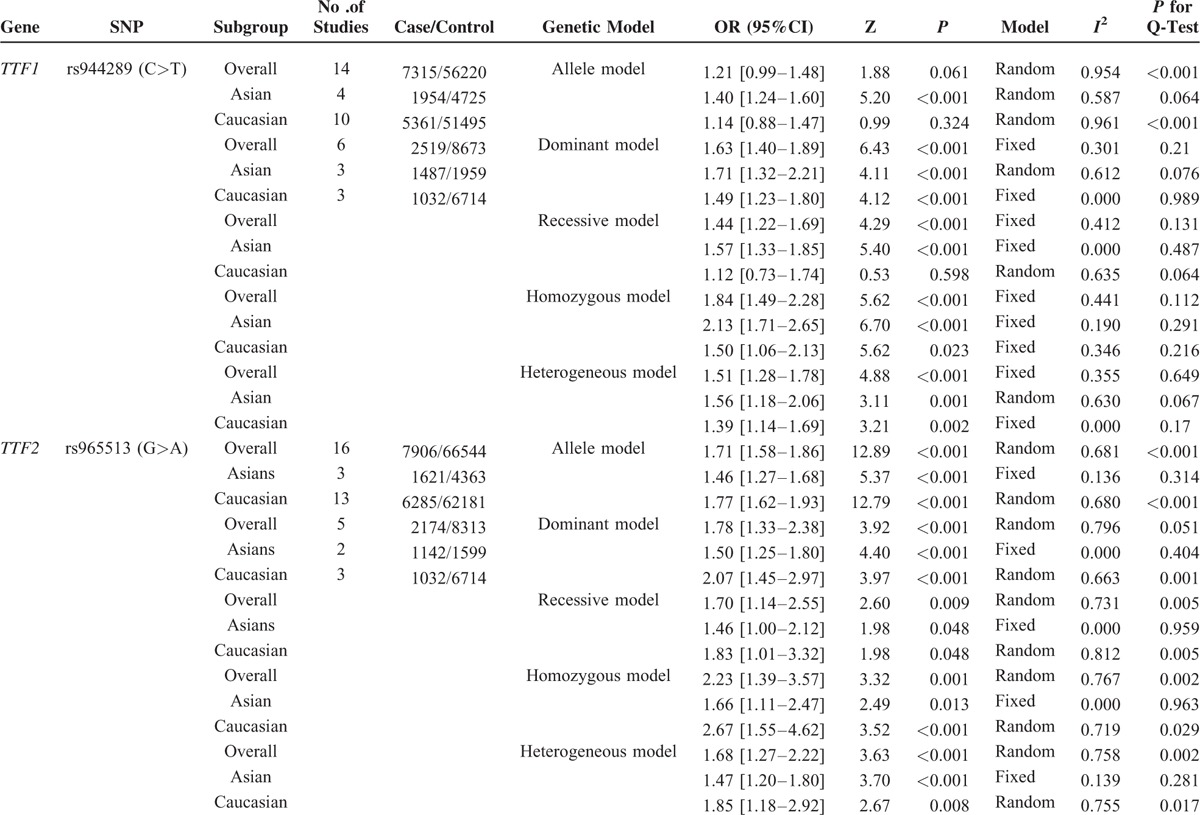
Results of Meta-analysis of the Associations Between rs944289/rs965513 and PTC

### Publication Bias

**FIGURE 3:**
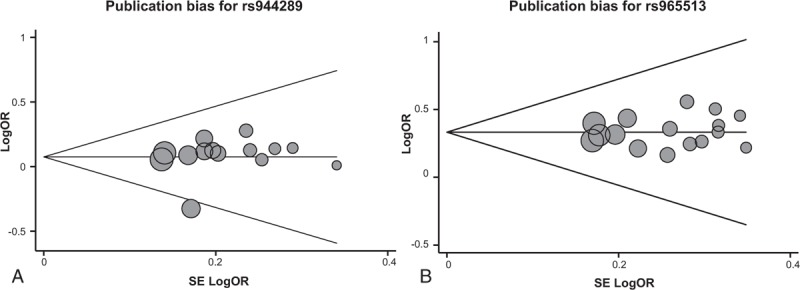
Funnel plot analysis to detect publication bias for rs944289 (a) and (b) rs965513.

Publication bias was assessed by the Funnel plot and Egger's test. No obvious asymmetry (*P* = 0.509, and *P* = 0.652 for rs944289 and rs965513, respectively) was revealed in the shape of the funnel plots (as shown in Figure 3).

## DISCUSSION

PTC, one of the most common malignant tumors, exits widely in the thyroid of adolescents.^35^ Up to now, the inaugural mechanism of PTC was considered as a complex process and was not clear, although it was acknowledged that genetic risk factors played an important role in the etiology of PTC. Recently, many researchers found that *TTF1* and *TTF2* could positively advance the risk of PTC in European population.^30,36–38^ rs944289/rs965513, which is the closest gene to *TTF1* and *TTF2* respectively, might be the best candidate for the association signals.^29,39^ The aim of the present study was to investigate the relationship between *TTF1* and *TTF2* polymorphisms and PTC susceptibility in Chinese population. As a result, variations of *TTF1* and *TTF2* were associated with an increased risk of PTC. Furthermore, subgroup analysis demonstrated that patients with MNG or no metastasis were more likely to have the risk of PTC. Additionally, our meta-analysis results revealed that the risk allele and genotype of *TTF1* and *TTF2* were the susceptible factors for developing PTC.

*TTF1*, which played an important role in differentiation of thyroid, expressed numerous tissues and cell types except thyroid, containing trachea, posterior pituitary, and hypothalamus.^40^*TTF2*, which located 57 kb downstream to rs965513, was also involved in differentiation of thyroid at the stage of embryo and formation of thyroid.^41^*TTF1* and *TTF2*, which were only found in thyroid together and they were all thyroid-specific transcription factors, regulated expression of the thyroid-specific genes, including genes of thyroglobulin and thyroxine.^42^ Studies found that expression changes or/and mutation of *TTF1* and *TTF2* had been related with many thyroid and non-thyroid diseases, such as BTLS, and Hirschsprung's disease.^14,20,32^ Subsequently, Homminga et al discovered that if TTF-1 integrated with receptor of T-cell or immunoglobulin heavy chain site, rearrangements of TTF-1 had positive correlation with the pathogenesis of hematopoietic malignancies.^43^ Recently, it is reported that *TTF1* and *TTF2* genes played an important role in tumor promotion, cell proliferation, differentiation, and migration, such as lung cancer and thyroid cancer.^44^ Though the pathogenesis mechanism is very complicated, the previous conclusion about the etiology between rs944289/ rs965513 and the risk of cancer may contribute to the further reconnoitering the pathogenesis of PTC.^2^

**FIGURE 4:**
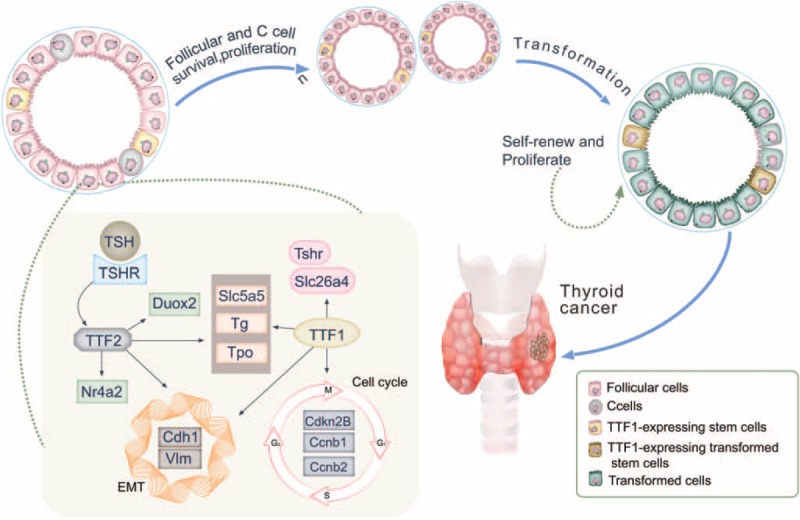
Mechanism between *TTF1* and *TTF2* gene variations and thyroid cancer.

Previous studies reported that *TTF1* regulates genes involved in the epithelial–mesenchymal transition (EMT) and cell cycle in thyroid follicular cells.^44^ And *TTF2* participates in EMT by regulating Cdh1 and Nr4a2 genes in thyroid follicular cells.^18^ Proteins including thyrotropin receptor (TSHR), thyroglobulin (TG), sodium/iodide cotransporter (SLC5A5), thyroid peroxidase (TPO), and dual oxidase 1 and 2 (DUOX1and DUOX2) play a critical role in the biosynthesis and secretion of thyroid hormones.^20^ We can speculate that *TTF1* and *TTF2* regulate several genes encoding these proteins and maintain the function of the thyroid (as shown in Figure 4). Meanwhile, researches indicated that transformed *TTF1* expressing stem cells produce thyroid cancer through self-renewing and proliferating, whereas a motile advantage of cancer cells might be conferred by *TTF2*. Mutations, epigenetic modifications and polymorphisms in *TTF1* and *TTF2* have been associated with thyroid cancer.

*TTF1*, which located at the promoter region and upstream of rs944289, can encode thyroglobulin (Tg) with co-activators, such as WW domain containing transcription regulator 1 (WWTR1).^45^ An auto regulator loop, which is activated by the binding sequences situated in the promoter region of *TTF1*, regulates *TTF1* own expression. The core binding locus is CAAG, and the missense variant of rs944289 can affect the binding sequence and result in amino acid substitution (valine instead of alanine at codon 339), which can induce multinodular goiter and PTC.^46^ Similarly, *TTF2* located 57 kb downstream to rs965513, and the promoter region included core sequence TTTGT.^47^ Gene mutation of rs965513 can result in *TTF2*, lost its function to bind DNA, and unable to stir transcription.^48^

Furthermore, results of stratification analyses revealed that the correction between rs944289 and rs965513 and increased PTC risk was more obvious in patients with MNG, indicating that MNG and gene mutation may promote the occurrence of PTC together. Interestingly, we discovered that the advanced susceptibility was more noticeable in subjects without metastasis, announcing that gene polymorphism of rs944289/rs965513 might be more primary than metastasis. Additionally, we demonstrated that the relationship between rs944289/rs965513 and PTC risk was significant in the overall population, including the Asians and the Caucasian. Nevertheless, the result was still controversial because the numbers of studies and sample size in Asian research were not ample to evaluate the association. Hence, to further substantiate the accuracy of the effect gene mutation of *TTF1* and *TTF2* on PTC susceptibility in the Asians, large-scale studies are needed.

Similarly, our study was limited in its effect estimation owing to small sample sizes for some comparisons. Moreover, these results in our study were just based on Chinese, which may not be applicable to other ethnicity. Besides, the present study was based on hospital and we couldn’t eliminate the possibility of selection bias of the subjects. Additionally, environmental factors as well as the interaction with individual genetic background, which are closely related to the pathogenesis of PTC, were not assessed in this study. Further studies of the risk effects and the functional impact of this polymorphism in further validation are in need.

In conclusion, we get that mutations of *TTF1* and *TTF2* are significantly associated with an increasing risk of PTC in Chinese. Furthermore, patients with MNG and no metastasis are more likely to suffer PTC. Through our meta-analysis, C/T variant of *TTF1* and G/A mutation of *TTF2* had a high correlation with PTC in the overall population. However, more detailed investigations and further large-scale studies on genetic functions to provide more conclusive and accurate evidence are required in the future.
